# Topological Design of Multi-Material Compliant Mechanisms with Global Stress Constraints

**DOI:** 10.3390/mi12111379

**Published:** 2021-11-10

**Authors:** Jinqing Zhan, Yifeng Li, Zhen Luo, Min Liu

**Affiliations:** 1 School of Mechatronics and Vehicle Engineering, East China Jiaotong University, Nanchang 330013, China; zhan_jq@126.com (J.Z.); liyifeng313@163.com (Y.L.); 2 School of Mechanical and Mechatronic Engineering, University of Technology Sydney, Ultimo, NSW 2007, Australia

**Keywords:** compliant mechanisms, multiple materials, topology optimization, global stress constraints, separable stress interpolation scheme

## Abstract

This paper presents an approach for the topological design of multi-material compliant mechanisms with global stress constraints. The element stacking method and the separable stress interpolation scheme are applied to calculate the element stiffness and element stress of multi-material structures. The output displacement of multi-material compliant mechanisms is maximized under the constraints of the maximum stress and the structural volume of each material. The modified *P*-norm method is applied to aggregate the local von Mises stress constraints for all the finite elements to a global stress constraint. The sensitivities are calculated by the adjoint method, and the method of moving asymptotes is utilized to update the optimization problem. Several numerical examples are presented to demonstrate the effectiveness of the proposed method. The appearance of the de facto hinges in the optimal mechanisms can be suppressed effectively by using the topology optimization model with global stress constraints, and the stress constraints for each material can be met.

## 1. Introduction

Compliant mechanisms often refer to a family of mechanisms that gain their mobility through the flexibility of some or all of its members due to the elastic body deformation [1]. Compared with the conventional rigid-body mechanisms, compliant mechanisms are featured with some unique benefits, such as easiness in fabrication, less wear and less backlash, requiring no lubrication and a built-in restoring force [2,3]. Due to these advantages and advances in advanced fabrication techniques, compliant mechanisms are already widely used in the field of precision engineering, bionic robots, intelligent structures and Micro-Electro-Mechanical Systems (MEMS) [4,5].

The methods of designing compliant mechanisms generally include two types. The first is the pseudo-rigid-body model-based method [6], which has been successfully adopted to design lumped compliant mechanisms with flexure hinges. The second is the topology optimization approach [7], which is mainly applied to design fully compliant mechanisms. Topology optimization of compliant mechanisms is a numerical process to obtain the best material distribution under a given amount of materials so that the prescribed mechanical performance can be achieved.

Ananthasuresh et al. [8] firstly introduced the homogenization method into the topological design of compliant mechanisms, which provided a new idea for designing compliant mechanisms. Thereafter, various topology optimization methods such as ground structure method [9], solid isotropic material with penalization [10,11], level set method [12,13], evolutionary structural optimization [14,15] and moving morphable components-based framework [16] were developed to design compliant mechanisms. Great progress has been gained in the topological design of compliant mechanisms. However, the topological design of compliant mechanisms is performed by using a single material in most studies. In practice, it is not always possible to use only one single material to design compliant mechanisms for achieving desired general performance. The advantages of different materials can be exploited fully to achieve comprehensive performance when multiple materials are adopted to design compliant mechanisms. It can give designers more freedom in designing advanced compliant mechanisms.

Therefore, the topological design of compliant mechanisms with multiple materials has attracted much attention. Yin et al. [17] applied a peak function material interpolation method to design multiple materials compliant mechanisms. Sigmund et al. [18] performed topological design of electrothermally driven actuators with multiple materials. Saxena et al. [19] proposed a method for the topological design of large-displacement and multi-material compliant mechanisms with multiple output ports. Wang et al. [20] used a color-level set method to design multi-material compliant mechanisms. Alonso et al. [21] used a sequential element rejection and admission method to perform topological design of multiple materials compliant mechanisms. Gaynor et al. [22] used a robust topology optimization method to design multi-material compliant mechanism. Zuo et al. [23] proposed an order multi-material isotropic material with penalization interpolation model for topological design of compliant mechanism without introducing any new variables. Wang et al. [24] applied a bi-level hierarchical optimization approach to perform topological design of compliant mechanisms with multiple materials.

Topology optimization of compliant mechanisms using no matter single material or multiple materials may be subject to de facto hinges, which usually results in stress concentration and poor fatigue performance. Great efforts have been made by many researchers to obtain hinge-free compliant mechanisms. Sigmund et al. [25] developed a class of morphology-based restriction methods to impose minimum length-scale constraints in order to eliminate the de facto hinges in the obtained compliant mechanisms. Poulsen [26] put forward a method for topological design of hinge-free compliant mechanisms by using a minimum length scale control. Yin et al. [27] used relative rotation constraints to suppress the appearance of the de facto hinges in topological design of compliant mechanisms. Zhou [28] used a hybrid discretization method to design compliant mechanisms, which can obtain hinge-free compliant mechanisms. For avoiding undesirable hinges, Luo et al. [29] developed a quadratic energy functional to control the geometric width of structural components. Wang et al. [30] put forward a method for topological design of hinge-free compliant mechanisms by using the intrinsic characteristic stiffness model. Zhu et al. [31] proposed a two-step elastic modeling method for designing compliant mechanisms, which can avoid de facto hinges.

More recently, De Leon et al. [32] adopted the stress-based topology optimization to design compliant mechanisms aiming at avoiding the appearance of the de facto hinges. Lopes et al. [33] performed topological design of compliant mechanisms with local von Mises stress constraints using the topological derivative concept, and the undesirable flexible hinges can be avoided. However, there are few studies about topology optimization of multi-material compliant mechanisms under stress constraints. Chu et al. [34] applied the stress penalty method for topology optimization design of multi-material compliant mechanism. The stress penalty term in the optimization objective is developed to control local stress constraints. Jeong et al. [35] used a separable stress interpolation scheme to solve the problem for topological design of continuum structure with multiple homogenous materials and stress constraints. Using local stress constraints, it gives rise to a topology optimization problem of multi-material compliant mechanisms with a large number of constraints. Consequently, a large computing time is required to solve the optimization problem. A large number of stress constraints can be aggregated into a global constraint by using a global stress constraints approach, and the computational efficiency can be greatly improved. However, no work has been conducted to study topological design of multiple materials compliant mechanisms using global stress constraints. 

The aim of this paper is to develop a new approach for topology optimization of compliant mechanisms with multiple materials under global stress constraints. The element stacking method and the separable stress interpolation scheme are applied to calculate the element stiffness and element stress of multi-material structures, respectively. The output displacement of multi-material compliant mechanisms is maximized under the constraints of the maximum stress and the structural volume of each material. The modified *P*-norm method is applied to aggregate the local von Mises stress constraints for all the finite elements to a global stress constraint. The sensitivities are calculated by the adjoint method, and the method of moving asymptotes is utilized to update the topology optimization problem.

The remainder of this paper is organized as follows. In Section 2, a new optimization model for topological design of multi-material compliant mechanisms with global stress constraints is proposed. In Section 3, the sensitivity analysis of the objective and constraints are described. In Section 4, several numerical examples are used to showcase the effectiveness of the proposed method. Conclusions are given in Section 5.

## 2. Optimization Problem

### 2.1. Material Interpolation Method

Within the existing multi-material interpolation methods such as extended solid isotropic material with penalization (SIMP) [9], the Young’s modulus of the optional materials with respect to design variables are generally interpolated and assigned to a single finite element. However, only one stress value per element can be evaluated in this approach. The standard SIMP-based multi-material interpolation methods may experience difficulty in solving the problem for topological design of multi-material compliant mechanisms with stress constraints. In addition, it may be difficult to directly use the standard multi-material topology optimization approaches when multiple different materials with different Poisson’s ratios are applied to design compliant mechanisms. Unlike the standard SIMP method, more than one element is juxtaposed on the same pixel, and a finite element is selected among elements having different material properties in the element stacking method [35]. Multiple stress values per element for each material can be calculated using the separable stress interpolation scheme based on the element stacking method [36]. Therefore, the element stacking method and the separable stress interpolation scheme are used to calculate the element stiffness and element stress for multi-material compliant mechanisms, respectively.

For finite element structural analysis of multi-material topology optimization problems, the element stacking method is applied to calculate the element stiffness as

(1) $$k_{e}\left(\rho \right)=\overset{NM}{\underset{i=1}{A}}\left[\rho_{e,i}^{p_{1}}\prod_{\begin{array}{l} j=1\\ j\neq i \end{array}}^{NM}\left[1-{\left(\rho_{e,j}\right)}^{p_{2}}k_{e,i}^{n}\right]\right]$$

Here, $k_{e}$ is the element stiffness matrix, ρ denotes the element density, namely, the design variables. $\rho_{e,i}$ and $\rho_{e,j}$ represent the design variables that the ith base material and the jth base material are assigned to the eth element, respectively. $k_{e,i}^{n}$ is nominal stiffness matrix of the eth element for ith base material, NM is the number of the base materials, $p_{1}$ and $p_{2}$ are the penalty exponents, which are set to 3 in this work, $\overset{NM}{\underset{i=1}{A}}$ denotes the pixel-level assembly operator of the element stiffness matrices.

Multiple stress values per element for each material can be calculated by the separable stress interpolation model

(2) $${\tilde{\sigma }}_{e,i}=\rho_{e,i}^{p_{3}}\sigma_{e,i}=\rho_{e,i}^{p_{3}}D_{i}^{n}Bu_{e}$$

where $\sigma_{e,i}$ is the stress vector of the eth element for ith base material, ${\tilde{\sigma }}_{e,i}$ is the relaxed stress vector and $p_{3}$ is the stress penalization factor. The motivation for introducing the relaxation method is to avoid the singularity phenomena, and it can make the optimization problem more tractable by generating a smooth feasible design space. There are various stress relaxation methods [37,38]. In this work, $p_{3}$ is set to be 0.5 as suggested in reference [38] and it works well. $D_{i}^{n}$ is the constitutive matrix of ith material, B is the strain-displacement matrix and $u_{e}$ is the element displacement vector.

For the plane stress problem, the constitutive matrix of the ith base material is defined as

(3) $$D_{i}^{n}=\frac{E_{i}}{1-\upsilon_{i}{}^{2}}\left[\begin{array}{ccc} 1 & \upsilon_{i} & 0\\ \upsilon_{i} & 1 & 0\\ 0 & 0 & \left(1-\upsilon_{i}\right)/2 \end{array}\right]$$

where $E_{i}$ and $\upsilon_{i}$ are the elastic modulus and the Poisson’s ratio for ith base material, respectively.

### 2.2. Global Stress Constraints

The von Mises stress of the eth element for ith material can be computed as

(4) $$\sigma_{e,i}^{\mathrm{vm}}=\sqrt{{\left(\sigma_{e,i}^{11}\right)}^{2}+{\left(\sigma_{e,i}^{22}\right)}^{2}+3{\left(\sigma_{e,i}^{12}\right)}^{2}-\sigma_{e,i}^{11}\sigma_{e,i}^{22}}={\left[{\left(\sigma_{e,i}\right)}^{T}V\sigma_{e,i}\right]}^{1/2}$$

Here, $\sigma_{e,i}^{11}$ and $\sigma_{e,i}^{22}$ are the stress components of the eth element for ith base material in the x and y direction, respectively. $\sigma_{e,i}^{12}$ is the element shear stress. V is the auxiliary matrix given by

(5) $$V=\left[\begin{array}{ccc} 1 & -1/2 & 0\\ -1/2 & 1 & 0\\ 0 & 0 & 3 \end{array}\right]$$

Similarly, the von Mises stress of the eth element for ith base material is penalized for intermediate design variable value

(6) $${\tilde{\sigma }}_{e,i}^{\mathrm{vm}}=\rho_{e,i}^{p_{3}}\sigma_{e,i}^{\mathrm{vm}}$$

where $\sigma_{e,i}^{\mathrm{vm}}$ is the nominal von Mises stress.

The design domain of compliant mechanisms is discretized into NE finite elements, and each element has NM local stress constraints. It means that the total number of local stress constraints NE×NM is too large and the computational demand is intensive. To improve the computational efficiency, the modified *P*-norm method [38,39] is applied to aggregate the local von Mises stress constraints for all the finite elements to a global stress constraint. The element volume is applied as an elemental scale factor that has the positive effect on convergence of the stress-constrained topology optimization problems [38]. Similarly, the element densities are used as a built-in scaling in this work. The *P*-norm stress measure of ith base material $\sigma_{\mathrm{PN},i}$ can be expressed as

(7) $$\sigma_{\mathrm{PN},i}={\left(\sum_{e=1}^{NE}\rho_{e,i}{\left({\tilde{\sigma }}_{e,i}^{\mathrm{vm}}\right)}^{p}\right)}^{\frac{1}{p}}$$

Here, p is the *P*-norm factor. When p approaches infinity, the *P*-norm stress measure is approximately equal to the maximum element stress. However, the higher value of p will increase the nonlinearity of the optimization problem. In accordance with our experience, when the factor p is set to 8, the method works well.

As the value of p cannot be too large, there is a large difference between the maximum stress and the *P*-norm stress measure. The adaptive constraint scaling factor [40] is applied to modify the *P*-norm stress measure in order to remedy the difference.

(8) $$\sigma_{\max,i}={\tilde{\sigma }}_{\mathrm{PN},i}\approx C_{i}\sigma_{\mathrm{PN},i}$$

where $\sigma_{\max,i}$ is the maximum stress value for ith base material and ${\tilde{\sigma }}_{\mathrm{PN},i}$ is the modified *P*-norm stress measure. $C_{i}$ is the scaling coefficient and can be defined as

(9) $$C_{i}^{{}^{\left(k\right)}}=\alpha_{i}{}^{\left(k\right)}\frac{\sigma_{\max,i}^{\left(k-1\right)}}{{\left(\sigma_{\mathrm{PN},i}\right)}^{\left(k-1\right)}}+\left(1-\alpha_{i}{}^{\left(k\right)}\right)C_{i}^{{}^{\left(k-1\right)}}$$

Here, ${\left(\sigma_{\mathrm{PN},i}\right)}^{\left(k-1\right)}$ and $\sigma_{\max,i}^{\left(k-1\right)}$ are the *P*-norm stress and the maximum stress value for ith base material at the *k*-1st iteration step, respectively. The parameter $\alpha_{i}{}^{\left(k\right)}$ is taken as 0.5, and $C_{i}^{{}^{\left(0\right)}}$ is set to 1.

### 2.3. The Topology Optimization Formulation

In this study, the maximization of the output displacement of multi-material compliant mechanisms is defined as the objective function. The maximum element stress and structural volume for each solid phase of materials are applied as the constraints. The model for the topology optimization of multi-material compliant mechanisms using global stress constraints can be expressed as

(10) $$\begin{array}{l} \max\;U_{\mathrm{out}}=L^{T}U\\ s.t.\;\sigma_{\max,}{}_{i}\approx C_{i}\sigma_{\mathrm{PN},i}\leq \sigma_{i}{}^{*}\left(i=1,2,\cdots ,NM\right)\\ \;V_{i}=\sum_{e=1}^{NE}\rho_{e,i}v_{0}\leq f_{i}{}^{*}V^{0}\left(i=1,2,\cdots ,NM,e=1,2,\cdots ,NE\right)\\ \;\;F=KU\\ \;\;\rho =\left[\begin{array}{ccc} \rho_{1,1} & \cdots & \rho_{NE,1}\\ \vdots & \vdots & \vdots \\ \rho_{1,NM} & \cdots & \rho_{NE,NM} \end{array}\right] \end{array}$$

where $U_{\mathrm{out}}$ represents the output displacement, U is the nodal displacement vector. L is a vector with value 1 at the desired output degree of freedom and with zeros at other all degrees of freedom. $\sigma_{\max,}{}_{i}$ is the maximum element stress for ith material, $\sigma_{i}{}^{*}$ is the allowable stress value for ith material, $v_{0}$ is the volume of the solid element, $V_{i}$ is the volume of the ith base material, $f_{i}{}^{*}$ is the allowable volume fraction for ith base material, $V^{0}$ is the total volume of design domain, K is the global stiffness matrix and F is the input load vector.

## 3. Sensitivity Analysis

The gradient-based optimization algorithm is used to solve the optimization problem with multiple constraints. The adjoint method is utilized to perform the sensitivities of the objective and constraints.

The objective $U_{\mathrm{out}}$ can be written as

(11) $$U_{\mathrm{out}}=L^{T}U+\eta^{T}(F-KU)$$

where η is the Lagrange multipliers.

The chain rule is applied to compute the sensitivity of the output displacement

(12) $$\frac{\partial U_{\mathrm{out}}}{\partial \rho_{e,i}}=\left[(L^{T}-\eta^{T}K)\frac{\partial U}{\partial \rho_{e,i}}-\lambda^{T}\frac{\partial K}{\partial \rho_{e,i}}U\right]$$

Let L=Kη. Then, we have

(13) $$\frac{\partial U_{\mathrm{out}}}{\partial \rho_{e,i}}=-\eta^{T}\frac{\partial K}{\partial \rho_{e,i}}U$$

The sensitivity of the volume constraint for ith material can be easily obtained as

(14) $$\frac{\partial V_{f}}{\partial \rho_{e,i}}=v_{0}$$

The sensitivity of the maximum stress for ith material can be expressed as

(15) $$\frac{\partial \sigma_{\max,i}}{\partial \rho_{e,i}}\approx C_{i}\frac{\partial \sigma_{\mathrm{PN},i}}{\partial \rho_{e,i}}=C_{i}\frac{\partial \sigma_{\mathrm{PN},i}}{\partial {\tilde{\sigma }}_{e,i}^{\mathrm{vm}}}\frac{\partial {\tilde{\sigma }}_{e,i}^{\mathrm{vm}}}{\partial \rho_{e,i}}$$

By taking the derivation of Equation (7), the term $\partial \sigma_{\mathrm{PN},i}/\partial {\tilde{\sigma }}_{e,i}^{\mathrm{vm}}$ can be obtained by

(16) $$\frac{\partial \sigma_{\mathrm{PN},i}}{\partial {\tilde{\sigma }}_{e,i}^{\mathrm{vm}}}={\left(\sum_{e=1}^{N}\rho_{e,i}{\left({\tilde{\sigma }}_{e,i}^{\mathrm{vm}}\right)}^{p}\right)}^{\frac{1}{p}-1}\rho_{e,i}{\left({\tilde{\sigma }}_{e,i}^{\mathrm{vm}}\right)}^{p-1}$$

By taking the derivation of Equation (6), the term $\partial {\tilde{\sigma }}_{e,i}^{\mathrm{vm}}/\partial \rho_{e,i}$ in Equation (15) can be obtained by

(17) $$\frac{\partial {\tilde{\sigma }}_{e,i}^{\mathrm{vm}}}{\partial \rho_{e,i}}=p_{3}\rho_{e,i}^{\left(p_{3}-1\right)}\sigma_{e,i}^{\mathrm{vm}}+\rho_{e,i}^{p_{3}}{\left(\frac{\partial \sigma_{e,i}^{\mathrm{vm}}}{\partial \sigma_{e,i}}\right)}^{T}\frac{\partial \sigma_{e,i}}{\partial \rho_{e,i}}$$

The term $\partial \sigma_{e,i}^{\mathrm{vm}}/\partial \sigma_{e,i}$ in Equation (17) can be expressed as

(18) $$\frac{\partial \sigma_{e,i}^{\mathrm{vm}}}{\partial \sigma_{e,i}}=\left[\begin{array}{l} \frac{\partial \sigma_{e,i}^{\mathrm{vm}}}{\partial \sigma_{e,i}^{11}}\\ \frac{\partial \sigma_{e,i}^{\mathrm{vm}}}{\partial \sigma_{e,i}^{22}}\\ \frac{\partial \sigma_{e,i}^{\mathrm{vm}}}{\partial \sigma_{e,i}^{12}} \end{array}\right]=\left[\begin{array}{c} \frac{1}{2\sigma_{e,i}^{\mathrm{vm}}}\left(2\sigma_{e,i}^{11}-\sigma_{e,i}^{22}\right)\\ \frac{1}{2\sigma_{e,i}^{\mathrm{vm}}}\left(2\sigma_{e,i}^{11}-\sigma_{e,i}^{22}\right)\\ \frac{3}{\sigma_{e,i}^{\mathrm{vm}}}\sigma_{e,i}^{12} \end{array}\right]$$

By substituting Equations (4) and (5) into Equation (18), the term $\partial \sigma_{e,i}^{\mathrm{vm}}/\partial \sigma_{e,i}$ can be simplified as

(19) $$\frac{\partial \sigma_{e,i}^{\mathrm{vm}}}{\partial \sigma_{e,i}}=\frac{V\sigma_{e,i}}{\sigma_{e,i}^{\mathrm{vm}}}$$

By taking the derivation of Equation (2), the term $\partial \sigma_{e,i}/\partial \rho_{e,i}$ can be expressed as

(20) $$\frac{\partial \sigma_{e,i}}{\partial \rho_{e,i}}=D_{i}^{n}B\frac{\partial u_{e}}{\partial \rho_{e,i}}$$

By taking the derivation of the equation F=KU, the following equation can be obtained while the loads are constant.

(21) $$\frac{\partial K}{\partial \rho_{e,i}}U+K\frac{\partial U}{\partial \rho_{e,i}}=0$$

The term $\partial u_{e}/\partial \rho_{e,i}$ in Equation (20) can be further written as

(22) $$\frac{\partial u_{e}}{\partial \rho_{e,i}}=\frac{\partial u_{e}}{\partial U}\frac{\partial U}{\partial \rho_{e,i}}=-\frac{\partial u_{e}}{\partial U}K^{-1}\frac{\partial K}{\partial \rho_{e,i}}U$$

where $\partial u_{e}/\partial U$ represents a transformation from local degrees of freedom to the global degrees of freedom.

By substituting Equation (22) into Equation (20), the term $\partial \sigma_{e,i}/\partial \rho_{e,i}$ can be further written as

(23) $$\frac{\partial \sigma_{e,i}}{\partial \rho_{e,i}}=-D_{i}^{n}B\frac{\partial u_{e}}{\partial U}K^{-1}\frac{\partial K}{\partial \rho_{e,i}}U$$

By substituting Equation (19) into Equation (17), Equation (17) can be further written as

(24) $$\frac{\partial {\tilde{\sigma }}_{e,i}^{\mathrm{vm}}}{\partial \rho_{e,i}}=p_{3}\rho_{e,i}^{\left(p_{3}-1\right)}\sigma_{e,i}^{\mathrm{vm}}+\rho_{e,i}^{p_{3}}{\left(\frac{V\sigma_{e,i}}{\sigma_{e,i}^{\mathrm{vm}}}\right)}^{T}\frac{\partial \sigma_{e,i}}{\partial \rho_{e,i}}$$

The term $\partial {\tilde{\sigma }}_{e,i}^{\mathrm{vm}}/\partial \rho_{e,i}$ in Equation (15) can be obtained by substituting Equation (23) into Equation (24)

(25) $$\frac{\partial {\tilde{\sigma }}_{e,i}^{\mathrm{vm}}}{\partial \rho_{e,i}}=p_{3}\rho_{e,i}^{\left(p_{3}-1\right)}\sigma_{e,i}^{\mathrm{vm}}-\rho_{e,i}^{p_{3}}\frac{\sigma_{e,i}^{T}V}{\sigma_{e,i}^{\mathrm{vm}}}D_{i}^{n}B\frac{\partial u_{e}}{\partial U}K^{-1}\frac{\partial K}{\partial \rho_{e,i}}U$$

Let $\eta =C_{i}{\left(\sum_{e=1}^{N}\rho_{e,i}{\left({\tilde{\sigma }}_{e,i}^{\mathrm{vm}}\right)}^{p}\right)}^{\frac{1}{p}-1}\rho_{e,i}{\left({\tilde{\sigma }}_{e,i}^{\mathrm{vm}}\right)}^{p-1}$. Then, Equation (15) can be simplified as

(26) $$\frac{\partial \sigma_{\max,i}}{\partial \rho_{e,i}}=\eta p_{3}\rho_{e,i}^{\left(p_{3}-1\right)}\sigma_{e,i}^{\mathrm{vm}}-\eta \rho_{e,i}^{p_{3}}\frac{\sigma_{e,i}^{T}V}{\sigma_{e,i}^{\mathrm{vm}}}D_{i}^{n}B\frac{\partial u_{e}}{\partial U}K^{-1}\frac{\partial K}{\partial \rho_{e,i}}U$$

The adjoint vector λ is obtained by the following equation.

(27) $$K\lambda =\frac{\eta \rho_{e,i}^{p_{3}}{\left(\frac{\partial u_{e}}{\partial U}\right)}^{T}B^{T}D_{i}^{n}V\sigma_{e,i}}{\sigma_{e,i}^{\mathrm{vm}}}$$

By substituting Equation (27) into Equation (26), we have

(28) $$\frac{\partial \sigma_{\max,i}}{\partial \rho_{e,i}}=(\eta p_{3}\rho_{e,i}^{\left(p_{3}-1\right)}\sigma_{e,i}^{\mathrm{vm}}-\lambda^{T}\frac{\partial K}{\partial \rho_{e,i}}U)$$

By taking the derivation of Equation (1), the term $\partial K/\partial {\tilde{\rho }}_{e,i}$ in Equation (28) can be obtained by

(29) $$\frac{\partial K}{\partial \rho_{e,i}}=\frac{\partial Κ}{\partial k_{e}}\frac{\partial k_{e}}{\partial \rho_{e,i}}=\frac{\partial Κ}{\partial k_{e}}\overset{NM}{\underset{i=1}{A}}\left[p_{1}\rho_{{}_{e,i}}^{\left(p_{1}-1\right)}\prod_{\begin{array}{l} j=1\\ j\neq i \end{array}}^{NM}\left[1-{\left(\rho_{{}_{e,j}}\right)}^{p_{2}}k_{e,i}^{n}\right]\right]$$

The mechanisms obtained by SIMP-based topology optimization method also experience the checker-board and the mesh-dependence phenomena. To resolve these numerical instability issues, the filtering technique [41] is applied to modify the sensitivities. The method of moving asymptotes (MMA) [42] is used to solve the topology optimization problem of multiple materials compliant mechanisms with a global stress constraint. The problem for stress-based topology optimization has a highly nonlinear behavior, which may easily lead to non-convergence in the iteration optimization. Therefore, an external move-limit of 0.1 is added to the MMA algorithm so that the maximum absolute difference between an asymptotic and the design variable is limited.

## 4. Numerical Examples

In this section, several numerical examples are presented to demonstrate the effectiveness of the proposed method. Both topological design of multi-material compliant mechanism with stress constraints and those without stress constraints will be performed to make comparison studies. In all the examples, the two base material properties are defined as follows: Young’s modulus of base material 1 and base material 2 are $E_{1}$ = 70 GPa and $E_{2}$ = 100 GPa, respectively, and the Poisson’s ratio of two base materials are $\mu_{1}=\mu_{2}=0.3$. The sensitivity filter radius $r_{\min}$ is set to 2.5, and all design variable $\rho_{e,i}$(*e* = 1, 2, … *NE*, *i* = 1, 2) are initialized to 0.5.

### 4.1. Displacement Inverter

**Figure 1:**
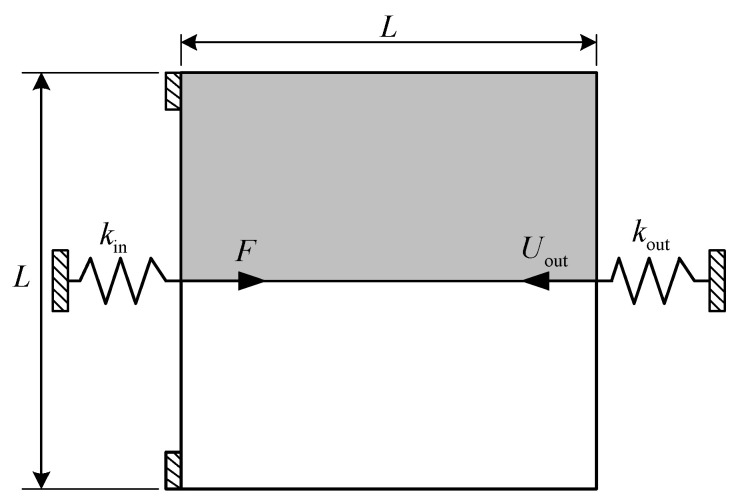
Design domain of compliant inverter.

For the first numerical example, the compliant inverter is to be designed, and the design domain with a square of 300 μm × 300 μm and a thickness of 10 μm is defined as Figure 1. The output port of compliant inverter is expected to generate an opposite output movement compared to the input port. The upper left edge and the lower left edge are fixed. The force loaded at the midpoint of the left side is 65 mN.

The input spring stiffness and the output spring stiffness are $k_{\mathrm{in}}$ = 2 × 10^3^ N/m and $k_{\mathrm{out}}$ = 1.6 × 10^3^ N/m, respectively. Note that, the input and output springs are artificial and they are used to model the interaction between the actuator and the mechanism or the workpiece and the mechanism, the detailed description can be found in the literature [43]. The allowable volume fraction of base material 1 and base material 2 are $f_{1}{}^{*}$ = 0.15 and $f_{1}{}^{*}$ = 0.125, respectively. Considering the symmetry, only the upper half of the design domain is considered and discretized to 100 × 50 bilinear quadrilateral elements.

**Figure 2:**
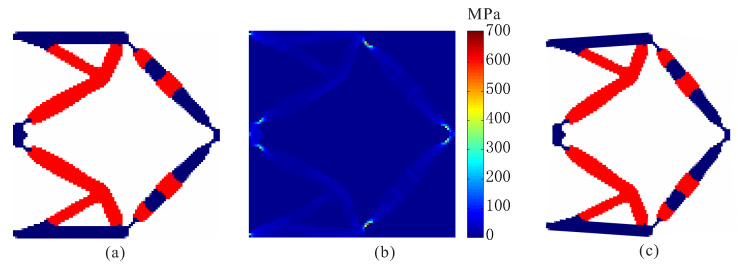
The results for topological design of multi-material compliant inverter without stress constraints. (**a**) Optimal topology; (**b**) stress distribution; (**c**) deformed configuration.

To verify the proposed method, topological design of two materials compliant mechanisms without stress constraints is performed firstly. In the topology optimization problem without stress constraints, the objective is developed as maximizing output displacement subject to volume constraints. The optimal layout of two-material compliant inverter obtained by the optimization model without stress constraints is shown in Figure 2. The red regions represent weak material and the blue regions denote strong material in the obtained inverter layout. As shown, one can find that de facto hinges obviously occur in the obtained compliant inverter, which cause stress concentrations. The maximum von Mises stresses are 329.432 MPa in weak material and 706.668 MPa in strong material, respectively. The output displacement of the obtained compliant inverter is 14.930 μm.

**Figure 3:**
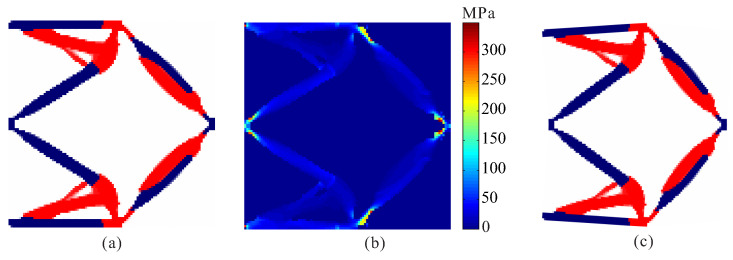
The results for topological design of multi-material compliant inverter with stress constraints ($\sigma_{1}^{*}$ = 275 MPa, $\sigma_{2}^{*}$ = 350 MPa). (**a**) Optimal topology; (**b**) stress distribution; (**c**) deformed configuration.

**Figure 4:**
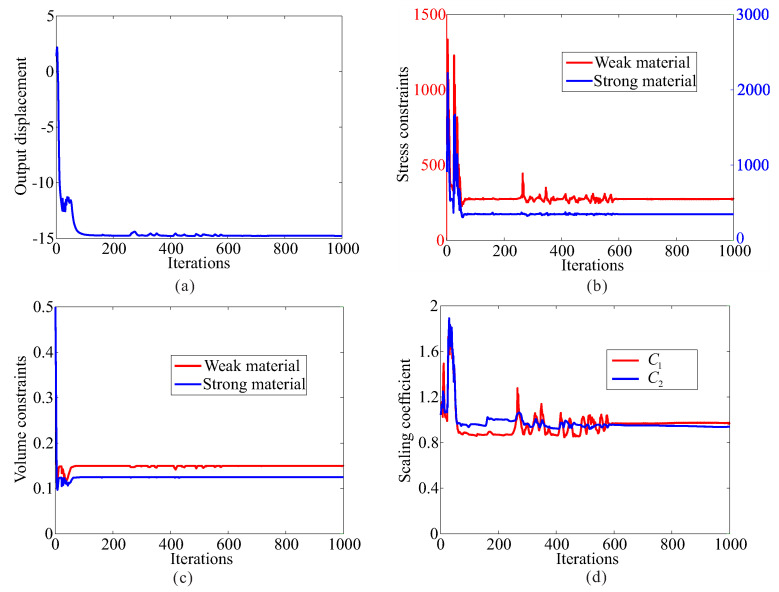
The convergence history for topological design of multi-material compliant inverter with global stress constraints ($\sigma_{1}^{*}$ = 275 MPa, $\sigma_{2}^{*}$ = 350 MPa). (**a**) Output displacement; (**b**) stress constraints; (**c**) volume constraints; (**d**) scaling coefficient.

Topological design of two-material compliant inverter with global stress constraints is performed. The allowable stresses are $\sigma_{1}{}^{*}$ = 275 MPa for the weak material and $\sigma_{2}{}^{*}$ = 350 MPa for the strong material, respectively. The optimal layout of the compliant inverter is obtained in Figure 3. Figure 4 shows the convergence histories of the output displacement, stress constraints, volume constraints and scaling coefficient for topological design of compliant inverter with global stress constraints. The optimization for this example converges after 1000 iterations. There will generally be relatively large oscillations in the initial stage because the volume of each material is reduced to meet the volume constraints. In addition, this is a multi-constraint optimization problem (two volume constraints and two stress constraints), in order to satisfy multiple constraints at the same time, oscillations may occur in the middle stage of optimization. The maximum von Mises stresses are 273.506 MPa in weak material and 349.561 MPa in strong material, respectively. One can notice that de facto hinges is effectively eliminated in the obtained compliant inverter and stress constraints for two materials can be met. By comparing stress distribution in Figure 2b and Figure 3b, one can see that the stress in the compliant inverter obtained by the proposed method is more uniformly distributed. It is demonstrated that the proposed method is effective for topological design of two materials inverter with global stress constraints. The output displacement of the obtained compliant inverter is 14.769 μm. The output displacement of the compliant inverter obtained by stress-based topology optimization is smaller than that of the inverter obtained by topology optimization without stress constraints. This is because the stress constraints limit the maximization of the output displacement to a certain degree.

**Figure 5:**
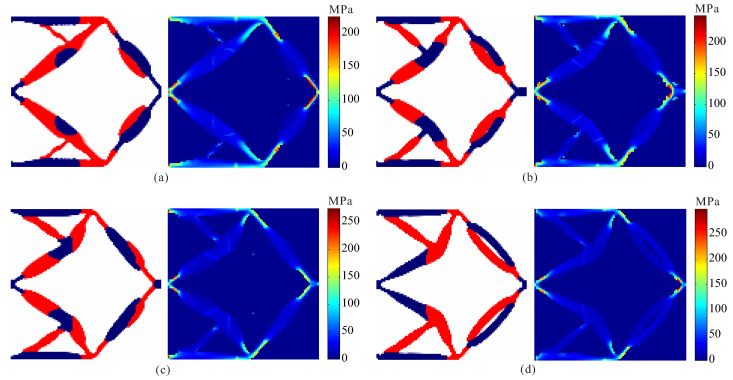
The results for topological design of multi-material compliant inverter under different stress constraint limits. (**a**) $\sigma_{1}^{*}$ = 150 MPa, $\sigma_{2}^{*}$ = 225 MPa; (**b**) $\sigma_{1}^{*}$ = 175 MPa, $\sigma_{2}^{*}$ = 250 MPa; (**c**) $\sigma_{1}^{*}$ = 200 MPa, $\sigma_{2}^{*}$ = 275 MPa; (**d**) $\sigma_{1}^{*}$ = 225 MPa, $\sigma_{2}^{*}$ = 300 MPa.

In order to investigate the effects of different stress constraints on the performance of optimal design, topological design of two-material compliant inverter is performed under different constraint limits for two materials. Comparison of optimal results obtained by topological design of multi-material compliant inverter with global stress constraints is given in Figure 5 and Table 1. From Table 1, one can find that the stress constraints for the weak and strong materials can be satisfied in all the case. It is observed that the optimal topologies of compliant inverter are different under different constraint limits in Figure 5. The hinge-free compliant inverters without de facto hinges can be obtained by using multi-material topology optimization model with global stress constraints. One can observe that as the stress constraint limits for two materials decreases, the output displacement of the obtained compliant inverters also lowers, and the stress is more uniformly distributed.

### 4.2. Compliant Gripper

**Figure 6:**
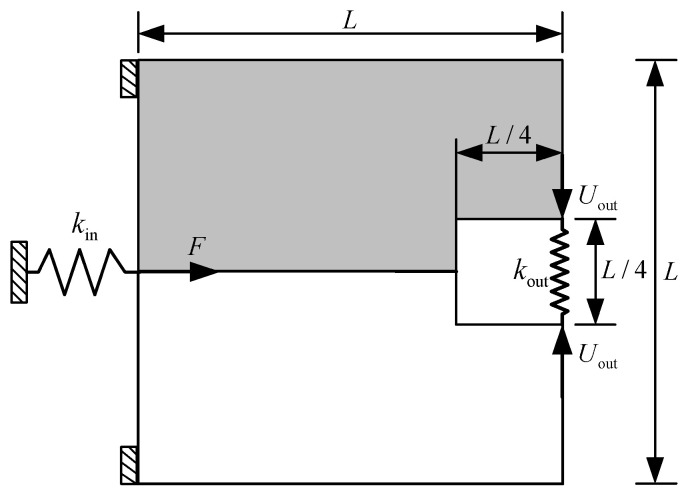
Design domain of compliant gripper.

In this example, the compliant gripper is to be designed, and the design domain with a square of 240 μm × 240 μm and a thickness of 10 μm is defined as Figure 6. The upper left edge and the lower left edge are fixed. The force loaded at the midpoint of the left side is 65 mN. The input spring stiffness and the output spring stiffness are $k_{\mathrm{in}}$ = 3 × 10^3^ N/m and $k_{\mathrm{out}}$ = 3 × 10^3^ N/m, respectively. The allowable volume fraction of weak material and strong material are $f_{1}{}^{*}$ = 0.120 and $f_{1}{}^{*}$ = 0.150, respectively. Considering the symmetry, only the upper half of the design domain is discretized by 80 × 40 bilinear quadrilateral elements for finite element analysis.

**Figure 7:**
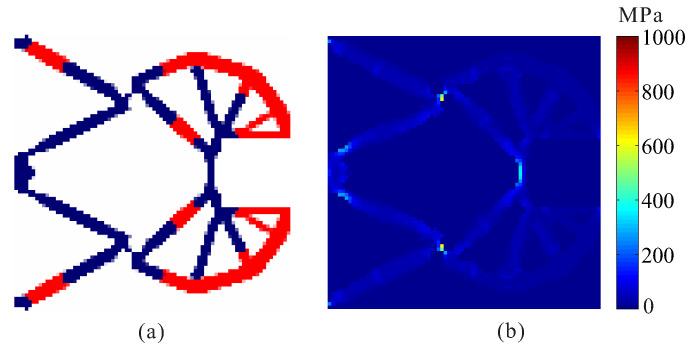
The results for topological design of multi-material compliant gripper without stress constraints. (**a**) Optimal topology; (**b**) stress distribution.

Topological design of two-material compliant gripper without stress constraints and with stress constraints are performed. The optimal layout of two materials compliant gripper obtained by the optimization model without stress constraints is shown in Figure 7. One can see that de facto hinges obviously occurs in the compliant gripper, causing stress concentrations. The maximum von Mises stresses are 227.924 MPa in weak material and 1001.756 MPa in strong material, respectively. The output displacement of the optimal gripper obtained by topology optimization without stress constraints is 7.341 μm.

**Figure 8:**
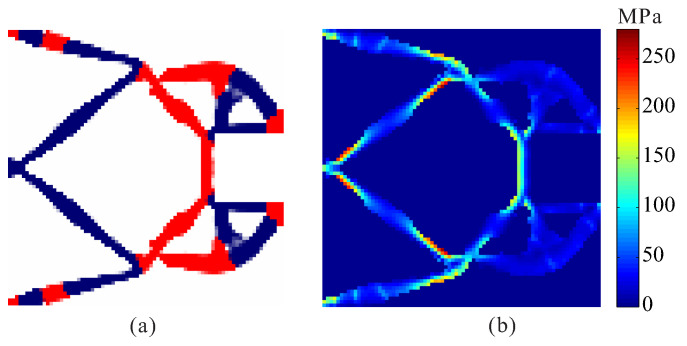
The convergence history for topological design of multi-material compliant gripper with global stress constraints ($\sigma_{1}^{*}$ = 180 MPa, $\sigma_{2}^{*}$ = 280 MPa). (**a**) Optimal topology; (**b**) stress distribution.

**Figure 9:**
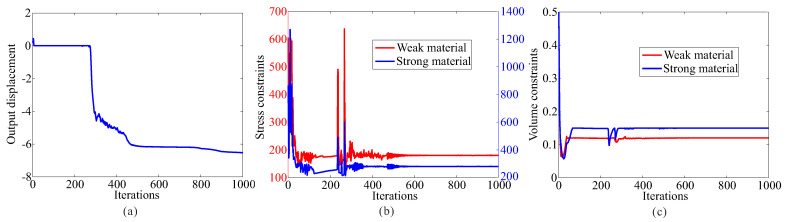
The convergence history for topological design of multi-material compliant gripper with global stress constraints ($\sigma_{1}^{*}$ = 180 MPa, $\sigma_{2}^{*}$ = 280 MPa). (**a**) Output displacement; (**b**) stress constraints; (**c**) volume constraints.

The allowable stresses are $\sigma_{1}{}^{*}$ = 180 MPa for the weak material and $\sigma_{2}{}^{*}$ = 280 MPa for the strong material, respectively. Figure 8 shows the optimal topology of multi-material compliant gripper obtained by the optimization model with global stress constraints. The maximum von Mises stresses in weak material and in strong material are 180.234 MPa and 279.946 MPa, respectively. Figure 9 shows the convergence histories of the output displacement, stress constraints and volume constraints for topological design of gripper with global stress constraints. The optimization for this example converges after 1000 iterations. There will generally be relatively large oscillations in the initial stage because the volume of each material is reduced to meet the volume constraints. In addition, this is a multi-constraint optimization problem, in order to satisfy multiple constraints at the same time, oscillations may occur in the middle stage of optimization. One can notice that the de facto hinges are effectively avoided in the compliant gripper and stress constraints for both weak material and strong material are met. By comparing the maximum stress in Figure 7b and Figure 8b, one can see that the stress in the compliant gripper obtained by the proposed method is more uniformly distributed. It is demonstrated that the proposed method is effective for topological design of two-material compliant gripper with global stress constraints. The output displacement of the obtained gripper is 6.526 μm. The output displacement of the gripper obtained by stress-based topology optimization is smaller than that of the gripper obtained by topology optimization without stress constraints. This is because the stress constraints limit the maximization of the output displacement to a certain degree.

**Figure 10:**
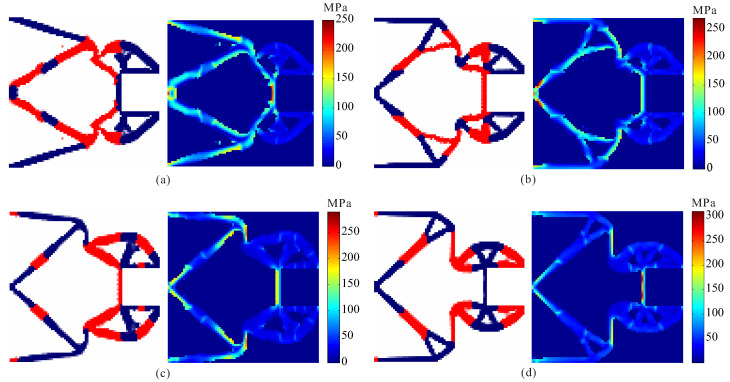
The results for topological design of multi-material compliant gripper under different stress constraint limits. (**a**) $\sigma_{1}^{*}$ = 150 MPa, $\sigma_{2}^{*}$ = 250 MPa; (**b**) $\sigma_{1}^{*}$ = 170 MPa, $\sigma_{2}^{*}$ = 270 MPa; (**c**) $\sigma_{1}^{*}$ = 190 MPa, $\sigma_{2}^{*}$ = 290 MPa; (**d**) $\sigma_{1}^{*}$ = 210 MPa, $\sigma_{2}^{*}$ = 310 MPa.

Topological design of two-material compliant gripper is performed under different constraint limits for two materials. Comparison of optimal results obtained by topological design of multi-material compliant gripper with global stress constraints is given in Figure 10 and Table 2. The optimal topologies of compliant grippers obtained by topology optimization under different constraint limits are different, and the stress constraints for each material are met in all the cases. It is observed that the hinge-free compliant grippers can be obtained by multi-material topology optimization model with global stress constraints. One can observe that as the stress constraint limits for two materials decreases, the output displacement of the obtained compliant grippers also lowers, and the stress is more uniformly distributed.

**Figure 11:**
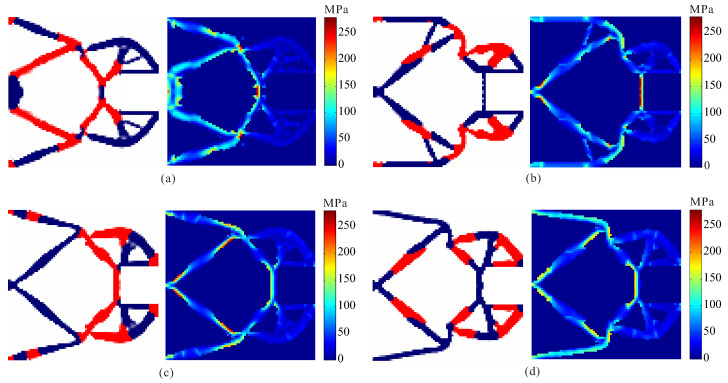
The results for topological design of multi-material compliant gripper under different output spring stiffness. (**a**) $k_{\mathrm{out}}$ = 1 × 10^3^ N/m; (**b**) $k_{\mathrm{out}}$ = 2 × 10^3^ N/m; (**c**) $k_{\mathrm{out}}$ = 3 × 10^3^ N/m; (**d**) $k_{\mathrm{out}}$ = 4 × 10^3^ N/m.

In order to investigate the effects of different output spring stiffness on the performance of optimized design, stress-based topology optimization of two materials compliant gripper is performed with different output spring stiffness. The allowable stresses are $\sigma_{1}{}^{*}$ = 180 MPa, $\sigma_{2}{}^{*}$ = 280 MPa for the weak material and the strong material, respectively. The optimal topologies and stress distribution of the obtained compliant grippers are shown in Figure 11. The optimal topologies of compliant grippers obtained by topology optimization under different output spring stiffness are different, and the stress constraints for each material are met in all the cases. It is observed that the output spring stiffness decreases, and the output displacement of the obtained compliant grippers increases, as shown in Table 3.

## 5. Conclusions

A method for topology optimization of multi-material compliant mechanisms with global stress constraints was put forward. The output displacement of multi-material compliant mechanisms is maximized under the constraints of the maximum stress and the structural volume of each material. The modified *P*-norm method is applied to aggregate the local von Mises stress constraints for all the finite elements to a global stress constraint. The method of moving asymptotes is utilized to update the topology optimization problem

Several numerical examples are presented to demonstrate the effectiveness of the proposed method. Comparing the results obtained by the model without stress constraints, the appearance of undesirable de facto hinges in the optimal mechanisms can be suppressed effectively by using the topology optimization model with global stress constraints, and the stress constraints for multiple materials are all met in all the cases. The optimal topologies of compliant mechanisms obtained by topology optimization with different constraint limits are different. As the stress constraint limits for two materials decreases, the output displacement of the obtained compliant mechanisms also lowers, and the stress is more uniformly distributed. The topologies of compliant mechanisms obtained by topological design under different output spring stiffness are also different. As the output spring stiffness decreases, the output displacement of the compliant mechanisms increases.

Compliant mechanisms may undergo large deformation during actual operation. Multi-material topology optimization of large-displacement compliant mechanisms with global stress constraints will be investigated in the near future.

## Figures and Tables

**Table 1:** Optimal results for compliant inverter under different stress constraint limits.

Stress Constraints		Output Displacement (μm)	Maximum Stress (MPa)	
$\sigma_{1}^{*}\;(\mathrm{MPa})$	$\sigma_{2}^{*}\;(\mathrm{MPa})$		Weak Material	Strong Material
150	225	13.361	149.538	224.976
175	250	13.941	172.011	243.847
200	275	14.376	200.065	275.011
225	300	14.587	224.986	299.837

**Table 2:** Optimal results for compliant gripper under different stress constraint limits.

Stress Constraints		Output Displacement (μm)	Maximum Stress (MPa)	
$\sigma_{1}^{*}\;(\mathrm{MPa})$	$\sigma_{2}^{*}\;(\mathrm{MPa})$		Weak Material	Strong Material
150	250	5.199	151.192	250.275
170	270	5.914	169.814	269.678
190	290	7.055	191.839	290.063
210	310	7.125	202.027	310.068

**Table 3:** Optimal results for compliant gripper under different output spring stiffness.

Output Spring Stiffness $k_{\mathrm{out}}$ (N/m)	Output Displacement (μm)	Maximum Stress (MPa)	
		Weak Material	Strong Material
1 × 10^3^	9.168	180.064	280.571
2 × 10^3^	7.579	179.894	279.709
3 × 10^3^	6.526	180.234	279.946
4 × 10^3^	5.239	177.928	280.065
